# Clinical phase II and III studies of an AS03‐adjuvanted H5N1 influenza vaccine produced in an EB66^®^ cell culture platform

**DOI:** 10.1111/irv.12755

**Published:** 2020-06-24

**Authors:** Masafumi Endo, Mitsuyoshi Tanishima, Kayo Ibaragi, Kenshi Hayashida, Tadashi Fukuda, Tetsuro Tanabe, Takeshi Naruse, Yoichiro Kino, Kohji Ueda

**Affiliations:** ^1^ KM Biologics Co., Ltd. (KM Biologics) Kumamoto Japan; ^2^ Kino Consulting Kumamoto Japan; ^3^ Kyushu University Fukuoka Japan

**Keywords:** AS03, EB66^®^ cells, H5N1 influenza

## Abstract

**Background:**

We have developed an AS03‐adjuvanted H5N1 influenza vaccine produced in an EB66^®^ cell culture platform (KD‐295).

**Objectives:**

In accordance with Japanese guidelines for development of pandemic prototype vaccines, the phase II study was conducted in a double‐blind, randomized, parallel‐group comparison study and the phase III study was conducted in an open‐label, non‐randomized, uncontrolled study.

**Methods:**

Healthy adult volunteers aged 20 ‐ 64 years enrolled in the phase II and III studies (N = 248 and N = 369) received KD‐295 intramuscularly twice with a 21‐day interval. After administration, immune response and adverse events were evaluated. In the phase II study, four different vaccine formulations were compared: MA (3.75 μg hemagglutinin [HA] antigen + AS03 adjuvant system), MB (3.75 μg HA + 1/2AS03), HA (7.5 μg HA + AS03), and HB (7.5 μg HA + 1/2AS03). In the phase III study, the MA formulation was further evaluated.

**Results:**

In the phase II study, all four vaccine formulations were well‐tolerated and no SAE related to vaccination were observed. The MA formulation was slightly more immunogenic and less reactogenic among the vaccine formulations. Therefore, the MA formulation was selected for the phase III study, and it was well‐tolerated and no serious adverse drug reactions were observed. The vaccine fulfilled the three immunogenicity criteria described in the Japanese guidelines.

**Conclusions:**

These data indicate that the MA formulation of KD‐295 was well‐tolerated and highly immunogenic and it can be considered a useful pandemic and pre‐pandemic influenza vaccine.

## 1 INTRODUCTION

The most recent influenza pandemic of 2009‐2010 remains fresh in our minds. Contrary to global expectations, the causative agent of the pandemic was an H1N1 virus. In the 2009 pandemic, various vaccines were used, including non‐adjuvanted and adjuvanted subvirion and whole virion vaccines.^1^ Among adults, the results of the vaccine use confirmed that even non‐adjuvanted vaccines were highly immunogenic. This is because there was cross‐reactivity in T helper epitopes between the H1N1 pandemic 2009 virus and previous seasonal H1N1 viruses.^2^ In Japan, the local vaccine manufacturers produced monovalent non‐adjuvanted split vaccine. At the same time, the Japanese government imported adjuvanted vaccines as a precaution in case of vaccine shortages, but many of these imported vaccines were left unused. However, pandemic threats, such as H5N1, have not disappeared and nobody knows what virus subtype will cause the next pandemic. At this moment, among the viruses with pandemic potential, viruses of avian origin, including the H7N9 subtypes, are a concern because of sporadic human infection.^3^ As same with H5N1 subtypes, and unlike the H1N1 pandemic 2009 virus, immunogenicity of those viruses is very low in humans, which may be related to predicted poor T‐cell immunogenicity.^4^

Another important condition of a pandemic vaccine is timely manufacturing. In the case of the 2009 pandemic, the causative virus was first isolated in April 2009 and a candidate vaccine virus was generated in May. Usually, seasonal influenza vaccines are produced from spring to summer in Japan; therefore, the transition of production from seasonal vaccine to pandemic vaccine was relatively smooth in 2009. If a pandemic occurs in a period outside of seasonal vaccine production in the egg vaccine platform, more time will be needed to start the vaccine manufacturing because of egg supply. Furthermore, because of the damage of chickens by highly pathogenic avian influenza, there is a risk that egg supply will be stopped. To address these issues, we have been developing an AS03‐adjuvanted vaccine using H5N1 influenza virus antigen derived from a duck cell line (EB66^®^). In the previous phase I study, we confirmed that the vaccine was well‐tolerated and elicited a broadly cross‐reactive antibody response.^5^ In this paper, we report further evaluation of AS03‐adjuvanted H5N1 influenza vaccine formulations produced in an EB66^®^ cell culture platform, KD‐295, in phase II and III studies to assess its immunogenicity and safety. In addition, phase II study data were registered and released in JapicCTI‐121788, and phase III study data were registered and released in JapicCTI‐121936.

## 2 MATERIALS AND METHODS

### 2.1 Study designs and subjects

The phase II study was conducted in adults between the ages of 20 and 64 years in a randomized, double‐blinded (all involved were blinded), comparative fashion from 2 April to 6 November 2012 to further assess immunogenicity and safety of the vaccine, and to determine the appropriate dosage to be evaluated in the phase III study. After selection of one formulation, the phase III study was performed from August 23, 2012 to March 10, 2013 in an unblinded, uncontrolled study enrolling adults between the age of 20 and 64 years.

In both studies, the selection criteria were healthy adults aged 20‐64 who agreed with written informed consent. Exclusion criteria included no history of H5N1 infection or vaccination. These studies were conducted in Tokyo, Osaka, and Kagoshima in Japan.

Prior to clinical studies, related documents such as the clinical trial protocol and informed consent form were reviewed by the IRB within each hospital. The studies were conducted in accordance with the Helsinki Declaration, GCP, and other relevant regulations. Written informed consent was obtained from participants prior to enrollment.

In the phase II study, four different vaccine formulations were evaluated: MA (3.75 μg HA + AS03), HA (7.5 μg HA + AS03), MB (3.75 μg HA + 1/2 AS03), and HB (7.5 μg HA + 1/2 AS03).

Regarding the allocation method to each group, first the sponsor distributed the investigational drugs allocated randomly to the study sites. The principal investigator or subinvestigator entered the information about subjects as of obtaining written informed consent and about the study sites into the Electronic Data Capture system (Medidata RaveTM). The investigational drug allocation system (Medidata BalanceTM) featured in the Electronic Data Capture system allocated the each subject to MA group, MB group, HA group, and HB group on 1:1:1:1 ratio by using a minimization method. Age, gender, stratification, study, and study sites were used as the adjustment factors.

In the phase III study, the MA formulation (3.75 μg HA + AS03) was evaluated based on the results of the phase I/II study. In both studies, the investigational vaccine KD‐295 was administered intramuscularly at a dose volume of 0.5 mL given twice at an interval of 21 ± 7 days.

### 2.2 Vaccines

KD‐295 was composed of separate vials consisting of hemagglutinin (HA) antigen and AS03 adjuvant (squalene, α‐tocopherol, and Tween 80) and mixed in equal amounts at the time of use. The vaccine virus strain used in this study was A/Indonesia/05/2005(H5N1)/PR8‐IBCDC‐RG2 strain belonging to Clade 2.1.3.2. Briefly, HA antigen was prepared as previously described5, by cultivating the vaccine virus in EB66^®^ cells, purifying the virus by sucrose density gradient centrifugation, inactivating with β‐propiolactone and ultraviolet light irradiation, and treating the virus particles with a surfactant.

### 2.3 Immunological evaluation

The immunogenicity evaluation protocols of the phase II and III studies were the same. Blood samples were taken before the first vaccination (Day 0), 21 days after the first vaccination (Day 21), and 21 days after the 2nd vaccination (Day 42). To evaluate the immune response to the vaccine strain, HI antibody and neutralizing antibody titers were measured. Measurements of HI antibody and neutralizing antibody titers were conducted with reference to a previously described method.^6^ For the HI test, both horse and chicken erythrocytes were used in the phase II study and only horse erythrocytes in the phase III study. For HI antibody, seroconversion rate (SCR), the geometric mean fold rise (GMFR), and the seroprotection rate (SPR) were calculated. SCR was the percentage of the subjects having an HI antibody titer of less than 1:10 prior to vaccination and 1:40 or higher after vaccination, or an HI titer before vaccination ≥1:10 and at least a 4‐fold increase in HI antibody titers after vaccination. SPR was defined as the percentage of participants who achieved HI titers of 1:40 or more. GMFR was defined as the ratio of change in GMT of HI antibody titers after the vaccination vs prior to the vaccination. The 95% confidence intervals on both sides of SCR and SPR were calculated based on the F‐distribution, and confidence interval of GMFR was calculated based on Student's t‐distribution.

For evaluation of immunogenicity of the vaccine, we followed the immunogenicity criteria of the Japanese guidelines for development of pandemic prototype vaccines (Japanese guidelines), which is identical to the immunogenicity criteria of the CHMP (Committee for Proprietary Medicinal Products) guidelines in place at the time the study was conducted (CPMP/BWP/214/96). In both studies, for the primary endpoint, we confirmed whether HI antibody (horse erythrocytes only) at Day 42 in a full analysis set (FAS) met the criteria of the Japanese guidelines (SCR >40%, GMFR >2.5, and SPR >70%). FAS was defined as a group of subjects with blood collected after at least one vaccination. The secondary endpoint of the both studies was percentage of subjects with a 4‐fold increase in neutralizing antibody titers at Day 42 in FAS. In addition, in the phase II study, we confirmed whether the chicken HI antibody at Day 42 met the criteria of the Japanese guidelines as a secondary endpoint.

In the phase II study, the target number of subjects was 50 in each group, for a total of 200, whereas the total number of enrolled subjects was 248, and the FAS included 246 subjects (62 for MA, 61 for HA, 63 for MB, 60 for HB). In the phase III study, the target number of subjects was 300, whereas the total number of subjects was 369, and the FAS included 364 subjects for MA. The target number of subjects in both studies was set according to the Japanese guidelines. Analyses were performed using SAS software version 9.2.

### 2.4 Safety evaluation

The safety evaluation protocols of the phase II and III studies were the same.

Using a health diary, solicited adverse events up to 7 days after each vaccination and unsolicited adverse events up to 21 days after each vaccination were recorded by subjects. Serious adverse events (SAE), important adverse events requiring cessation of vaccination (IAE) or adverse events including autoimmune diseases, and other inflammatory or autoimmune pathogenic neurological diseases (pIMDs: potential immune‐mediated diseases) were recorded during the study for another 6 months after the last vaccination (Day 0‐Day 201). Adverse events were graded in three levels; (a) mild (event that is easily tolerable, accompanied by only slight discomfort and does not interrupt daily activities); (b) moderate (event that interrupts daily activities because of discomfort); and (c) severe (event that makes daily activities impossible). Solicited adverse events were divided into two types, local and systemic adverse events. Solicited local adverse events included injection site erythema, swelling, and induration that occurred in the period following administration of the investigational drug until 6 days after administration. All solicited local adverse events were reactions at the injection site and so were categorized as adverse drug reactions. Solicited systemic adverse events included pyrexia, headache, fatigue, arthralgia, myalgia, chills, and hyperhidrosis that occurred in the period following administration of the investigational drug until 6 days after administration. The potential causal relationship between vaccination and other symptoms or events was determined by the investigator, and if a relationship was recognized, adverse events were categorized as adverse drug reactions. Statistical analyses were performed using SAS software version 9.2.

### 2.5 Study population

Demographic data in the FAS of the phase II and III studies are shown in Table 1. The mean ± SD for age in the FAS was 39.1 ± 11.1 years and 37.8 ± 11.2 years overall in the phase II and III studie,s respectively. No population imbalance was observed among the groups.

**TABLE 1:** Demographic data in the phase II and III studies full analysis set (FAS)

Item	Statistics	Phase II									Phase III
		MA group	HA group	MB group					HB group	Total	MA group
Number of analyzed subjects		62	61	63					60	246	364
Age (year)	Mean	39.5	39.3	38.9					38.7	39.1	37.8
	Standard deviation	12.9	10.4	10.9					10.4	11.1	11.2
	Minimum	20	22	21					20	20	20
	Median	36.0	39.0	38.0					37.0	37.0	37.5
	Maximum	64	63	62					64	64	64
Number of subjects （Incidence %）											
Age	41 y and over	37 (59.7)	35(57.4)	36 (57.1)			35 (58.3)			143 (58.1)	222 (61.0)
	Less than 40 y	25 (40.3)	26 (42.6)		27 (42.9)		25 (41.7)			103 (41.9)	142 (39.0)
Sex	Male	27 (43.5)	28 (45.9)		29 (46.0)		28 (46.7)			112 (45.5)	179 (49.2)
	Female	35 (56.5)	33 (54.1)		34 (54.0)		32 (53.3)			134 (54.5)	185 (50.8)
Ethnicity	Japanese	62 (100.0)	61 (100.0)		63 (100.0)		60 (100.0)			246 (100.0)	364 (100.0)
	Other	0 (0.0)	0 (0.0)		0 (0.0)		0 (0.0)			0 (0.0)	0(0.0)
Medical history	No	58 (93.5)	56 (91.8)		57 (90.5)		55 (91.7)			226 (91.9)	342 (94.0)
	Yes	4 (6.5)	5 (8.2)		6 (9.5)		5 (8.3)			20 (8.1)	22(6.0)
Underlying disease	No	48 (77.4)	50 (82.0)			54 (85.7)		52 (86.7)		204(82.9)	285 (78.3)
	Yes	14 (22.6)	11 (18.0)			9 (14.3)		8 (13.3)		42 (17.1)	79 (21.7)
Allergy	No	56 (90.3)	51 (83.6)			57 (90.5)		54 (90.0)		218 (88.6)	289(79.4)
	Yes	6 (9.7)	10 (16.4)			6 (9.5)		6 (10.0)		28 (11.4)	75 (20.6)

Note

MA group; 3.75 μg HA + AS03. HA group; 7.5 μg HA + AS03. MB group; 3.75 μg HA + 1/2 AS03. HB group; 7.5 μg HA + 1/2 AS03.

## 3 RESULTS

### 3.1 Immunogenicity

HI antibody against the vaccine strain was measured using horse and chicken erythrocytes in the phase II study. In the measurement using horse erythrocytes, the HI antibody response to the vaccine strain after the second vaccination (Day 42) in the FAS fulfilled all three criteria of immunogenicity described in the guidelines in all groups. For measurement using chicken erythrocytes, GMFR fulfilled the guideline criteria in all groups; however, SCR in the MB group and SPR in all groups did not fulfill the criteria. Since horse erythrocytes had higher sensitivity at HI antibody measurement, in the phase III study, only the horse erythrocyte assay was used. Results of the HI test for both studies are shown in Table 2. Although there was no large difference in the immunogenicity between the different vaccine formulations in the phase II study, the point estimate for GMFR was higher in the groups given the vaccine with the standard amount of AS03 (HA, MA) vs a half dose (MB, HB). The MA formulation was evaluated in the phase III study considering the safety data described later. In the phase III study, the MA formulation was highly immunogenic and it fulfilled all three criteria described in the guidelines.

**TABLE 2:** Conformance of the parameters of the HI antibody (horse RBCs and chicken RBCs) response to the second vaccination (Day 42) with the three immunogenicity criteria of the guideline

Point estimate (95%CI)	Phase II								Phase III	
	MA group		HA group		MB group		HB group		MA group	
Number of subjects	60		59		61		60		364	
Horse red blood cells										
Seroconversion rate >40%	100.0% (94.0%‐100.0%)	*	100.0% (93.9%‐100.0%)	*	100.0% (94.1%‐100.0%)	*	98.3% (91.1%‐100.0%)	*	100.0% (99.0‐100.0)	*
					28.56 (24.69‐33.04)					
Geometric mean fold rise >2.5	33.90 (28.82‐39.88)	*	40.48 (34.39‐47.64)	*		*	30.55 (25.44‐36.70)	*	43.73 (41.15‐46.47)	*
					(24.69‐33.04) 100.0% (94.1%‐100.0%)					
Seroprotection rate >70%	100.0% (94.0%‐100.0%)	*	100.0% (93.9%‐100.0%)	*		*	98.3% (91.1%‐100.0%)	*	100.0% (99.0‐100.0)	*
GMT	169.5 (144.1‐199.4)		202.4 (171.9‐238.2)		142.8 (123.5‐165.2)		152.8 (127.2‐183.5)		220.3 (207.3‐234.1)	
Chicken red blood cells										
Seroconversion rate >40%	55.0% (41.6%‐67.9%)	*	64.4% (50.9%‐76.4%)	*	39.3% (27.1%‐52.7%)		56.7% (43.2%‐69.4%)	*	‐	
Geometric mean fold rise >2.5	6.20 (4.84‐7.96)	*	7.37 (5.80‐9.36)	*	4.38 (3.54‐5.42)	*	6.20 (4.93‐7.81)	*	‐	
Seroprotection rate >70%	55.0% (41.6%‐67.9%)		64.4% (50.9%‐76.4%)		39.3% (27.1%‐52.7%)		56.7% (43.2%‐69.4%)		‐	
GMT	31.0 (24.2‐39.8)		37.3 (29.4‐47.2)		21.9 (17.7‐27.1)		31.0 (24.6‐39.1)		‐	

Note

Strain measured: A/Indonesia/05/2005(H5N1). Vaccine strain: A/Indonesia/05/2005(H5N1). Subjects analyzed: a group of subjects who was collected blood after the second vaccination. Confidence interval (seroconversion rate, seroprotection rate):lower limit and upper limit of the exact 95% two‐sided confidence interval based on F‐distribution. Confidence interval (rate of change in GMT): lower limit and upper limit of the 95% two‐sided confidence interval based on Student's t‐distribution. MA group; 3.75 μg HA + AS03. HA group; 7.5 μg HA + AS03. MB group; 3.75μg HA+1/2 AS03. HB group; 7.5μg HA+1/2 AS03.

* Fulfilled the immunogenicity criteria of the guideline.

GMT of neutralizing antibody and percentage of subjects with a 4‐fold increase in neutralizing antibody titers against vaccine strains after the 1st and 2nd vaccination in FAS are shown in Table 3. Both in the phase II and III studies, after the 1st dose, the seroconversion rates were 20‐30% and rose to nearly 100% after the second vaccination (Day 42). At the same time, GMT increased markedly in all groups after the second vaccination (Day 42).

**TABLE 3:** Changes in geometric mean titer of neutralizing antibody to the vaccine strain

Timing	Phase II								Phase III	
	MA group		HA group		MB group		HB group		MA group	
	GMT (Confidence interval)	Rates of antibody rise of 4‐fold or higher (%) [Confidence interval (%)]	GMT (Confidence interval)	Rates of antibody rise of 4‐fold or higher (%) [Confidence interval (%)]	GMT (Confidence interval)	Rates of antibody rise of 4‐fold or higher (%) [Confidence interval (%)]	GMT (Confidence interval)	Rates of antibody rise of 4‐fold or higher (%) [Confidence interval (%)]	GMT (Confidence interval)	Rates of antibody rise of 4‐fold or higher (%) [Confidence interval (%)]
Number of analyzed subjects	62		61		63		60		364	
Before vaccination (Day 0)	5.1 (4.9‐5.2)	－	5.1 (4.9‐5.2)	－	5.0	－	5.0	－	5.0	－
After the 1^st ^vaccination (Day 21)	9.8 (8.0‐12.0)	27.4 (16.9‐40.2)	13.1 (10.7‐16.2)	39.3 (27.1‐52.7)	8.7 (7.3‐10.3)	20.6 (11.5‐32.7)	9.5 (7.8‐11.7)	21.7 (12.1‐34.2)	10.2 (9.5‐11.1)	27.5 (22.9‐32.4)
After the 2^nd ^vaccination (Day 42)	234.3 (182.5‐300.7)	100.0 (94.0‐100.0)	265.2 (205.0‐343.0)	98.3 (90.9‐100.0)	171.3 (130.9‐224.1)	100.0 (94.1‐100.0)	211.1 (162.1‐274.9)	98.3 (91.1‐100.0)	301.1 (275.4‐329.2)	99.7 (98.5‐100.0)

Strain measured: A/Indonesia/05/2005(H5N1). Vaccine strain: A/Indonesia/05/2005(H5N1).Subjects analyzed: FAS.GMT: geometric mean antibody titer. Confidence interval: lower limit and upper limit of the 95% two‐sided confidence interval based on Student's t‐distribution. MA group; 3.75 μg HA + AS03. HA group; 7.5 μg HA + AS03. MB group; 3.75 μg HA + 1/2 AS03. HB group; 7.5 μg HA + 1/2 AS03.

### 3.2 Safety

Adverse events and adverse drug reactions occurring during the study period (Day 0‐Day 201) are summarized in Table 4. Two SAEs (thyroid cancer and acute abdomen) and one pIMD (pasuda disease) occurred in the phase 3 trial, but in all cases a causal relationship with vaccination was denied. Through both studies, no serious adverse drug reactions (death, SAE, IAE, and pIMD related to vaccination) were reported. Among unsolicited adverse drug reactions, injection‐site pruritus showed the highest incidence in both studies. The majority of cases of injection site pruritus were grade 1. grade 3 unsolicited adverse drug reactions occurred in one case (dehydration) in the phase II study the MA group and in two cases (positional vertigo and malaise) in the phase III study. The incidence rates were low.

**TABLE 4:** Safety summary of the phase II and III studies

Classification of adverse events	Investigation periods	Phase II												Phase III		
		MA group			HA group			MB group			HB group			MA group		
		Number of subjects with events			Number of subjects with events			Number of subjects with events			Number of subjects with events			Number of subjects with events		
			Incidence (%) (95%CI)			Incidence (%) (95%CI)			Incidence (%) (95%CI)			Incidence (%) (95%CI)			Incidence (%) (95%CI)	
Number of analyzed subjects		62			62			63			61			369		
Adverse events																
Death	Day 0‐Day 201	0			0			0			0			0		
Serious adverse events	Day 0‐Day 201	0			0			0			0			2	0.5	(0.1‐1.9)
Significant adverse events	Day 0‐Day 201	0			0			0			0			0		
Potential immune‐mediated diseases	Day 0‐Day 201	0			0			0			0			1	0.3	(0.0‐1.5)
Solicited local adverse events	Day 0‐Day 6, Day 21‐Day 27	54	87.1	(76.1‐94.3)	52	83.9	(72.3‐92.0)	52	82.5	(70.9‐90.9)	48	78.7	(66.3‐88.1)	330	89.4	(85.8‐92.4)
Solicited systemic adverse events	Day 0‐Day 6, Day 21‐Day 27	43	69.4	(56.3‐80.4)	46	74.2	(61.5‐84.5)	45	71.4	(58.7‐82.1)	35	57.4	(44.1‐70.0)	247	66.9	(61.9‐71.7)
Unsolicited adverse events	Day 0‐Day 42	22	35.5	(23.7‐48.7)	20	32.3	(20.9‐45.3)	25	39.7	(27.6‐52.8)	22	36.1	(24.2‐49.4)	124	33.6	(28.8‐38.7)
	Day 43‐Day 201	0			0			0			0			1	0.3	(0.0‐1.5)
	Day 0‐Day 201	22	35.5	(23.7‐48.7)	20	32.3	(20.9‐45.3)	25	39.7	(27.6‐52.8)	22	36.1	(24.2‐49.4)	125	33.9	(29.1‐39.0)
Adverse drug reactions																
Death	Day 0‐Day 201	0			0			0			0			0		
Serious adverse events	Day 0‐Day 201	0			0			0			0			0		
Significant adverse events	Day 0‐Day 201	0			0			0			0			0		
Potential immune‐mediated diseases	Day 0‐Day 201	0			0			0			0			0		
Solicited local adverse events	Day 0‐Day 6, Day 21‐Day 27	54	87.1	(76.1‐94.3)	52	83.9	(72.3‐92.0)	52	82.5	(70.9‐90.9)	48	78.7	(66.3‐88.1)	330	89.4	(85.8‐92.4)
Solicited systemic adverse events	Day 0‐Day 6, Day 21‐Day 27	41	66.1	(53.0‐77.7)	44	71.0	(58.1‐81.8)	45	71.4	(58.7‐82.1)	34	55.7	(42.4‐68.5)	245	66.4	(61.3‐71.2)
Unsolicited adverse events	Day 0‐Day 42	18	29.0	(18.2‐41.9)	16	25.8	(15.5‐38.5)	15	23.8	(14.0‐36.2)	13	21.3	(11.9‐33.7)	99	26.8	(22.4‐31.7)
	Day 43‐Day 201	0			0			0			0			0		
	Day 0‐Day 201	18	29.0	(18.2‐41.9)	16	25.8	(15.5‐38.5)	15	23.8	(14.0‐36.2)	13	21.3	(11.9‐33.7)	99	26.8	(22.4‐31.7)

Note

Sbjects analyzed: safety analysis set. Study period: Day 0 ‐Day 201. Confidence intervalexact 95% two‐sided confidence interval based on F‐distribution. MA group; 3.75 μg HA + AS03. HA group; 7.5 μg HA + AS03. MB group; 3.75 μg HA + 1/2 AS03. HB group; 7.5 μg HA + 1/2 AS03.

Table 5 and Table 6 show the incidence rates of solicited local adverse events, solicited systemic adverse events, and solicited systemic adverse drug reactions occurring during the investigation periods (Day 0‐Day 6 and Day 21‐Day 27). In the phase II study, the rate of injection site pain, 70%‐90%, was the highest among the solicited local adverse events in all groups. The incidence rates of other solicited local adverse events were between 10% and 30%. The incidence rates of injection site pain and other solicited local adverse events in the phase III study were similar to those in the phase II study. In addition, the incidence rate of grade 3‐solicited adverse events was low.

**TABLE 5:** Incidence of solicited local adverse events (Day 0‐Day 6 and Day 21‐Day 27)

	No. of subjects with the event				
	Incidence(%)(Confidenceinterval)				
	Phase II				Phase III
	MA group	HA group	MB group	HB group	MA group
Number of analyzed subjects	62	62	63	61	369
Solicited local adverse events	54	52	52	48	330
	87.1% (76.1‐94.3)	83.9% (72.3‐92.0)	82.5% (70.9‐90.9)	78.7% (66.3‐88.1)	89.4% (85.8‐92.4)
Pain					
Total	53	52	49	45	320
	85.5% (74.2‐93.1)	83.9% (72.3‐92.0)	77.8% (65.5‐87.3)	73.8% (60.9‐84.2)	86.7% (82.8‐90.0)
Grade 3	0	0	0	0	0
	0%	0%	0%	0%	0%
Erythema					
Total	18	19	10	12	126
	29.0% (18.2‐41.9)	30.6% (19.6‐43.7)	15.9% (7.9‐27.3)	19.7% (10.6‐31.8)	34.1% (29.3‐39.2)
Grade 3	1	0	1	0	8
	1.6% (0.0‐8.7)	0%	1.6% (0.0‐8.5)	0%	2.2% (0.9‐4.2)
Swelling					
Total	17	15	8	10	106
	27.4% (16.9‐40.2)	24.2% (14.2‐36.7)	12.7% (5.6‐23.5)	16.4% (8.2‐28.1)	28.7% (24.2‐33.6)
Grade 3	1	0	0	0	4
	1.6% (0.0‐8.7)	0%	0%	0%	1.1% (0.3‐2.8)
Induration					
Total	17	14	10	11	82
	27.4% (16.9‐40.2)	22.6% (12.9‐35.0)	15.9% (7.9‐27.3)	18.0%（9.4‐30.0)	22.2% (18.1‐26.8)
Grade 3	1	0	0	0	0
	1.6% (0.0‐8.7)	0%	0%	0%	0%

Note

Analysis set: Safety analysis set. Period of investigation: Day 0‐Day 6 and Day 21 ‐Day 27.Item: Adverse event. MedDRA/J (Ver15.1). MA group; 3.75 μg HA + AS03. HA group; 7.5 μg HA + AS03. MB group; 3.75 μg HA + 1/2 AS03. HB group; 7.5 μg HA + 1/2 AS03.

**TABLE 6:** Incidence of solicited systemic adverse events and solicited systemic adverse drug reactions (Day 0‐Day 6 and Day 21‐Day 27)

PT	No. of subjects with the event				
	Incidence(%)(Confidenceinterval)				
	Phase II				Phase III
	MA group	HA group	MB group	HB group	MA group
Number of analyzed subjects	62	62	63	61	369
	43	46	45	35	248
Solicited systemic adverse events	69.4% (56.3‐80.4)	74.2% (61.5‐84.5)	71.4% (58.7‐82.1)	57.4% (44.1‐70.0)	67.2% (62.2‐72.0)
Pyrexia					
Total	8	17	4	3	86
	12.9% (5.7‐23.9)	27.4% (16.9‐40.2)	6.3% (1.8‐15.5)	4.9% (1.0‐13.7)	23.3% (19.1‐28.0)
Grade 3	4	1	0	0	6
	6.5% (1.8‐15.7)	1.6% (0.0‐8.7)	0%	0%	1.6% (0.6‐3.5)
Headache					
Total	21	26	26	20	132
	33.9% (22.3‐47.0)	41.9% (29.5‐55.2)	41.3% (29.0‐54.4)	32.8% (21.3‐46.0)	35.8% (30.9‐40.9)
Grade 3	1	0	0	0	1
	1.6% (0.0‐8.7)	0%	0%	0%	0.3% (0.0‐1.5)
Fatigue					
Total	36	36	27	27	157
	58.1% (44.8‐70.5)	58.1% (44.8‐70.5)	42.9% (30.5‐ 56.0)	44.3% (31.5‐57.6)	42.5% (37.4‐47.8)
Grade 3	1	0	0	0	0
	1.6% (0.0‐8.7)	0%	0%	0%	0%
Arthralgia					
Total	18	17	11	8	97
	29.0% (18.2‐41.9)	27.4% (16.9‐40.2)	17.5% (9.1‐29.1)	13.1% (5.8‐24.2)	26.3% (21.9‐31.1)
Grade 3	0	0	0	0	1
	0%	0%	0%	0%	0.3% (0.0‐1.5)
Myalgia					
Total	23	23	21	21	124
	37.1% (25.2‐50.3)	37.1% (25.2‐50.3)	33.3% (22.0‐46.3)	34.4% (22.7‐47.7)	33.6% (28.8‐38.7)
Grade 3	0	0	0	0	0
	0%	0%	0%	0%	0%
Chills					
Total	13	17	7	5	93
	21.0% (11.7‐33.2)	27.4% (16.9‐40.2)	11.1% (4.6‐21.6)	8.2% (2.7‐18.1)	25.2% (20.9‐30.0)
Grade 3	1	0	0	0	1
	1.6% (0.0‐8.7)	0%	0%	0%	1.3% (0.0‐1.5)
Hyperhidrosis					
Total	4	7	13	5	44
	6.5% (1.8‐15.7)	11.3% (4.7‐21.9)	20.6% (11.5‐32.7)	8.2% (2.7‐18.1)	11.9% (8.8‐15.7)
Grade 3	0	0	0	0	0
	0%	0%	0%	0%	0%
Solicited systemic adverse drug reactions	41	44	45	34	245
	66.1% (53.0‐77.7)	71.0% (58.1‐81.8)	71.4% (58.7‐82.1)	55.7% (42.4‐68.5)	66.4% (61.3‐71.2)
Pyrexia					
Total	8	17	4	3	85
	12.9% (5.7‐23.9)	27.4% (16.9‐40.2)	6.3% (1.8‐15.5)	4.9% (1.0‐13.7)	23.0% (18.8‐27.7)
Grade 3	4	1	0	0	6
	6.5% (1.8‐15.7)	1.6% (0.0‐8.7)	0%	0%	1.6% (0.6‐3.5)
Headache					
Total	20	25	25	20	131
	32.3% (20.9‐45.3)	40.3% (28.1‐53.6)	39.7% (27.6‐52.8)	32.8% (21.3‐46.0)	35.5% (30.6‐40.6)
Grade 3	1	0	0	0	1
	1.6% (0.0‐8.7)	0%	0%	0%	0.3% (0.0‐1.5)
Fatigue					
Total	36	34	27	25	156
	58.1% (44.8‐70.5)	54.8% (41.7‐67.5)	42.9% (30.5‐ 56.0)	41.0% (28.6‐54.3)	42.3% (37.2‐47.5)
Grade 3	1	0	0	0	0
	1.6% (0.0‐8.7)	0%	0%	0%	0%
Arthralgia					
Total	18	17	11	7	96
	29.0% (18.2‐41.9)	27.4% (16.9‐40.2)	17.5% (9.1‐29.1)	11.5% (4.7‐22.2)	26.0% (21.6‐30.8)
Grade 3	0	0	0	0	1
	0%	0%	0%	0%	0.3% (0.0‐1.5)
Myalgia					
Total	22	23	21	20	122
	35.5% (23.7‐48.7)	37.1% (25.2‐50.3)	33.3% (22.0‐46.3)	32.8% (21.3‐46.0)	33.1% (28.3‐38.1)
Grade 3	0	0	0	0	0
	0%	0%	0%	0%	0%
Chills					
Total	12	17	7	5	93
	19.4% (10.4‐31.4)	27.4% (16.9‐40.2)	11.1% (4.6‐21.6)	8.2% (2.7‐18.1)	25.2% (20.9‐30.0)
Grade 3	1	0	0	0	1
	1.6% (0.0‐8.7)	0%	0%	0%	0.3% (0.0‐1.5)
Hyperhidrosis					
Total	4	7	13	5	44
	6.5% (1.8‐15.7)	11.3% (4.7‐21.9)	20.6% (11.5‐32.7)	8.2% (2.7‐18.1)	11.9% (8.8‐15.7)
Grade 3	0	0	0	0	0
	0%	0%	0%	0%	0%

Note

Analysis set: Safety analysis set. Period of investigation: Days 0‐6 and Days 21‐27.Item: Adverse event, Adverse drug reaction. MedDRA/J (Ver15.1)

Of the solicited systemic adverse events and solicited systemic adverse drug reactions, that with the highest incidence was fatigue, which was expressed in 40%‐60% of patients in each group. The next highest were headache and myalgia (30%‐40%). In addition, the incidence rate of grade 3‐solicited systemic adverse events and solicited systemic adverse drug reactions was low.

## 4 DISCUSSION

The phase II and phase III clinical studies revealed that MA formulation of KD‐295 (3.75 μg HA + AS03) was well‐tolerated and highly immunogenic and that no serious adverse drug reactions were observed. Therefore, the MA formulation can be considered as a useful pandemic and pre‐pandemic influenza vaccine.

Although most human cases of avian influenza to date have been associated with direct contact with infected birds, as causative agents for the next pandemic, viruses including H5, H7, and H9 subtypes are still of concern as viruses with pandemic potential.^7^ Among them, the H5 subtype was the first target for vaccine development, and since then many types of vaccine have been developed. The first developed H5N1 vaccine was a non‐adjuvanted split vaccine, and it was reported that two doses of 90 μg HA of the vaccine‐induced neutralization antibody titers reaching 1:40 or greater in 54 percent of study subjects.^8^ Non‐adjuvanted and adjuvanted whole virion vaccines were then developed, and their immunogenicity in humans with 7.5‐15 μg HA antigen dose was much better than that of the split vaccine.^9^ We also conducted a clinical study with an egg‐derived, alum‐adjuvanted whole virion H5N1 vaccine.^6^ However, although the vaccine was immunogenic, it could not meet one of the three criteria of CHMP guidelines. Therefore, we decided to develop a more immunogenic vaccine with a platform other than chicken eggs to have flexibility in the vaccine production.

As a result of immunological evaluation in the phase II study, all vaccine groups fulfilled the three required criteria described in the Japanese guidelines based on the HI antibody titers measured using horse erythrocytes after administration of two doses of the vaccine.

When vaccine strain derived from avian influenza virus such as H5N1 is used as an antigen, HI antibody titers' measurement using horse erythrocytes has more sensitive in detection of the antigen than using chicken erythrocytes.^10^ This is why HI antibody titers measured using not chicken, but horse erythrocytes were used for primary evaluation.

Although not statistically significant, GMFR was higher in the groups given the vaccine with standard AS03 (HA, MA) dose. Although all the vaccine formulations were well‐tolerated, the MA formulations were less incidence of solicited systemic adverse events (pyrexia, headache, and chills) than the HA formulations. Therefore, the MA formulation was selected to be evaluated in the phase III study.

In the phase III study, the MA formulation containing a standard dose of AS03 and 3.75 μg HA antigen was further confirmed to be highly immunogenic and fulfilled the all criteria of the Japanese guidelines. The results of clinical studies, including those of the present study, confirm that the AS03 adjuvant is potent and the antigen dose could be reduced to 3.75 μg HA. Regarding an H5N1 vaccine with antigen derived from EB66^®^ cells and formulated with AS03, Schuind et al^11^ reported similar results to ours in their clinical phase I study. Chada et al^12^ conducted a meta‐analysis and concluded that vaccines with emulsion‐type adjuvants could induce broad cross‐clade antibodies and are suitable for stockpiling. Feldstein et al^13^ compared human immunogenity data of several H5N1 vaccines and concluded that adjuvanted H5N1 vaccines induced high theoretical efficacy and that AS03‐adjuvanted vaccine was more immunogenic than MF59‐adjuvanted vaccine. It has also been confirmed that the AS03 could increase immunogenicity of H7N1, H7N9, and H9N2 antigens.^14^, ^15^, ^16^, ^17^ Based on these data, this study has limitations because it does not directly compare KD‐295 with other vaccines, but KD‐295 appears to be more effective than other licensed H5N1 vaccines.

Marichal et al proposed that alum‐adjuvant induces neutrophil migration and cell death, and subsequently DNA released from the host cell activates innate immunity as DAMPs.^18^ AS03‐adjuvant reportedly activates not only innate immunity, but also adaptive immunity comprehensively, induces production of various cytokines, and contributes to enhancing the antigen‐specific antibody production of B cells.^19^, ^20^ It also reported that α‐tocopherol plays an important role in these immune responses. In fact, omission of α‐tocopherol from AS03 modified the innate immune response and lead to lower antibody responses.^19^ Therefore, at this time, vaccines with emulsion‐type adjuvant, including AS03 with α‐tocopherol, would be the promising choice for both pre‐pandemic and pandemic avian influenza vaccines.

When compared with the safety profile of our alum‐adjuvanted H5N1 whole virion vaccine,^6^ the greatest difference between the two vaccines is local (injection site) pain. With the alum‐adjuvanted vaccine, 10%‐45% of participants reported local pain after administration of the vaccine, whereas 70%‐80% of subjects reported it with the AS03‐adjuvanted vaccine. Local pain is a common adverse drug reaction with emulsion‐type adjuvanted of vaccines.^9^ However, it is self‐limiting and leads to no further complication. The incidence rates of other local and systemic adverse events observed in the current studies were generally higher than those of alum‐adjuvanted vaccines; however, most events were grades 1 and 2, and the highest rate of grade 3 was pyrexia yet at only 1.6% which led to no further complication. In general, as was the case in the phase I study, the vaccine was well‐tolerated in both the phase II and III studies.

Finally, for the possible next pandemic, global cooperation will be essential for an effective response. For this purpose, the WHO established the Pandemic Influenza Preparedness (PIP) framework for the sharing of influenza viruses and access to vaccines and other benefits. The WHO PIP documents^21^ state that member states should urge vaccine manufacturers to set aside a portion of each production cycle of pandemic influenza vaccine for use by developing countries. Therefore, in the event of a pandemic, several kinds of vaccines will be distributed at the same time in those countries. As described previously, immunogenicity of the licensed pandemic vaccines is varied, and vaccines with the emulsion‐type adjuvants are the most immunogenic. Leroux‐Roels et al^22^ reported that priming with AS03‐adjuvanted H5N1 influenza vaccine improves the immune response of a heterologous booster vaccination. They also reported that priming with non‐adjuvanted vaccine appears to inhibit the response to subsequent vaccination; therefore, KD‐295 also should be evaluated in combination of several vaccines with different immunogenicity. In conclusion, although it remains necessary to evaluate in pediatric and elderly populations, the MA (3.75 μg HA + AS03) formulation was well‐tolerated and highly immunogenic. KD‐295 can be considered as a useful pandemic and pre‐pandemic influenza vaccine. In addition, KD‐295 is currently approved in Japan.

## CONFLICT OF INTEREST

Kohji Ueda received a fee from KAKETSUKEN, currently KM Biologics Co., Ltd., for the implementation of this study. Yoichiro Kino, which belongs to Kino Consulting, was an employee of KAKETSUKEN at the time of the study period. The other authors have no conflicts of interest of declare.
